# Vitamin D Levels in Patients Presenting to a Rheumatology Clinic in Germany: Associations with Patient Characteristics and Season

**DOI:** 10.3390/nu17111893

**Published:** 2025-05-31

**Authors:** Martin Feuchtenberger, Magdolna Szilvia Kovacs, Axel Nigg, Arne Schäfer

**Affiliations:** 1 Rheumatologie, MVZ MED BAYERN OST, 84489 Burghausen, Germany; 2 Medizinische Klinik und Poliklinik II, University Hospital Würzburg, 97080 Würzburg, Germany; 3 Diabetes Zentrum Mergentheim, 97980 Bad Mergentheim, Germany

**Keywords:** vitamin D, vitamin D deficiency, epidemiology, rheumatology, electronic health records, seasons

## Abstract

**Background**: High rates of vitamin D deficiency have been reported in population-based studies, including those conducted in Germany. The goal of this study was to evaluate vitamin D levels and associated factors in a clinical cohort of German patients presenting to a rheumatology clinic. **Methods**: We conducted a retrospective observational study of electronic health record data from patients presenting to a rheumatology clinic in southern Germany. Data included demographic characteristics and vitamin D levels as measured by the Elecsys^®^ Vitamin D total III assay (Roche). Associations between vitamin D levels and patient characteristics were evaluated by Pearson correlation analyses, *t*-tests, and multiple regression analyses. We also explored seasonal changes. **Results**: A total of 4979 patients were included; 3230 (64.9%) were female and the mean (standard deviation [SD]) age was 53.6 (15.2) years. The mean (SD) vitamin D level was 27.4 (14.0) ng/mL (range, 3–240 ng/mL). Overall, 1540 (30.9%) had vitamin D levels in the deficient range (<20 ng/mL), 1774 (35.6%) had sufficient vitamin D (20 to 30 ng/mL), 1597 (32.1%) had optimal vitamin D levels (>30 to 70 ng/mL), and 68 (1.4%) had levels >70 ng/mL. Lower vitamin D levels were significantly associated with younger age, male sex, and higher body mass index. Mean levels were significantly lower during winter months and the percentages of patients with vitamin D deficiency were higher. **Conclusions**: Our data indicate that low levels of vitamin D are common in clinical cohorts, particularly in men, younger adults, overweight individuals, and during winter months. Patient education and/or supplementation may help to address this issue and potentially improve patient health.

## 1. Introduction

Vitamin D is a multi-functional biomolecule and hormone that plays a vital role in human health and influences both skeletal and non-skeletal functions [1]. The two major forms of vitamin D, vitamin D2 (ergocalciferol) and vitamin D3 (cholecalciferol), require activation through hydroxylation in the liver, forming 25-hydroxyvitamin D (25[OH]D), followed by further conversion, primarily in the kidneys, to the active form, 1,25-dihydroxyvitamin D (1,25[OH]_2_D). This active form binds to the vitamin D receptor and modulates gene expression in various tissues [1,2]. Commercial serum assays for vitamin D measure 25(OH)D, as this compound reflects the total vitamin D reservoir [3]. For some patients, including patients with kidney disease, vitamin D-resistant rickets, and granulomatous conditions, assays that measure 1,25(OH)_2_D may provide supplementary information on vitamin D status [4,5].

In addition to playing a key role in maintaining bone health, vitamin D has other diverse health effects, particularly on the innate and adaptive immune systems [1]. Vitamin D deficiency (serum levels <20 ng/mL [50 nmol/L]) [6] has been associated with a wide variety of health disorders, including bone disorders such as osteoporosis, autoimmune diseases such as rheumatoid arthritis, respiratory infections, cardiovascular disease, type 2 diabetes, asthma and other allergic diseases, and neuropsychiatric disorders [2,5]. Although vitamin D supplementation is effective in increasing serum levels [7], there is mixed evidence for the beneficial effects of vitamin D supplementation on non-skeletal outcomes [1]. Possible explanations for this variability include issues with study design, differences among patients in factors known to affect vitamin D levels, such as individual metabolism, age, and body mass index (BMI), and known seasonal variations in vitamin D levels [1]. Despite the inconsistent results observed with vitamin D supplementation, a meta-analysis of randomized controlled trials of vitamin D use in adults, mostly in women older than 70 years, found that the rate of mortality was significantly reduced in individuals receiving vitamin D3 supplementation compared with controls [8], suggesting that vitamin D supplementation has the potential to result in clinically relevant effects.

Because sun exposure is a key factor in the synthesis of endogenous vitamin D, vitamin D deficiency is common in many countries with northern latitudes, including Germany. A study of data collected by the German Health Interview and Examination Survey for Adults, which was conducted between 2008 and 2011, reported that 61.5% of individuals had serum 25(OH)D levels <20 ng/mL, indicating vitamin D deficiency [9]; this figure was later revised to 56.0% based on standardization of the assay used [10]. This rate is markedly higher than the 28.9% vitamin D deficiency rate reported in the United States [11] and the 20.4% rate reported in Canada [12], but consistent with rates reported for other countries with similar northern latitudes (40 to 60° N; 40.6% to 50.0%) [13].

In addition to sun exposure, vitamin D levels are influenced by multiple additional factors, including age, sex, use of vitamin D supplements, BMI, skin pigmentation, metabolic differences, sunscreen use, and chronic illnesses, particularly liver or kidney diseases [11,14]. Many of these factors are likely to be different in a clinical population compared with the overall population. In particular, medications, pre-existing illnesses, reduced mobility/sun exposure, and autoimmune/inflammatory manifestations are likely to vary between population-wide and clinical samples. To gain further insights into vitamin D status in a large clinical population, we conducted a retrospective analysis of vitamin D levels in a large cohort of patients seen at a rheumatology center for the evaluation of rheumatologic complaints.

Rheumatology disorders span a broad spectrum of inflammatory and non-inflammatory conditions. The most common inflammatory rheumatology disorder is rheumatoid arthritis, which affects approximately 0.5% to 1% of individuals in Europe and Northern America [15], while osteoarthritis is the most common form of arthritis and affects an estimated 7.6% of individuals worldwide [16]. Vitamin D levels may have an especially important impact in rheumatology patients [17], as by definition, these patients have musculoskeletal and/or immune disorders. In addition, medications that increase the risk of osteoporosis, particularly glucocorticoids, are frequently used in this patient population. Although several studies have been published on vitamin D supplementation in defined rheumatology populations, such as patients with rheumatoid arthritis [17], there is limited information on vitamin D levels in patients referred for the evaluation of rheumatologic complaints during routine clinical care. The objective of this study was to fill this gap in the literature by providing contemporary data on the vitamin D status of a large clinical population presenting to a rheumatology clinic in Germany and to investigate associations between vitamin D levels, patient variables, and season.

## 2. Materials and Methods

### 2.1. Study Design

This retrospective cohort study was based on electronic health record (EHR) data collected from adult and pediatric patients who presented for the first time to a single, large secondary care center specializing in rheumatology in southern Germany (Burg-hausen) between 1 January 2021 and 31 December 2024 and had blood tests for vitamin D levels performed as part of routine clinical care. At this center, vitamin D measurement is performed only at initial presentation, unless there are clinical signs indicating the need for further investigation. Subsequent vitamin D tests for a given patient, if any, were not included in this study. Variables of interest included data on patient characteristics, renal status, and vitamin D assay results (see below). This study was approved by the Institutional Review Board of Würzburg University and received a waiver for individual patient consent due to the retrospective design and use of de-identified patient data (#207/21-me). All research activities were conducted in accordance with the ethical principles outlined in the Declaration of Helsinki.

### 2.2. Renal Status

Renal function was assessed by estimated glomerular filtration rate (eGFR in mL/min/1.73 m^2^) based on serum creatinine as measured by the Jaffe assay and adjusted for age and sex [18]. The levels of kidney function based on eGFR were derived from the Kidney Disease: Improving Global Outcomes (KDIGO) practice guidelines for the management of chronic kidney disease (CKD) [19].

### 2.3. Vitamin D Assay

Vitamin D levels were determined by the Elecsys^®^ Vitamin D total III assay (Roche Diagnostics GmbH, Mannheim, Germany), an electrochemiluminescence binding assay that measures the levels of 25(OH)D (both 25-hydroxyvitamin D2 and 25-hydroxyvitamin D3) in human serum and plasma [20]. Assays were performed at the clinic site.

Although there are many varying definitions for different categories of vitamin D levels [21], for the purposes of this analysis, deficient vitamin D levels were defined as <20 ng/mL (<50 nmol/L). The cut-offs for other categories used in this study are shown in Table 1.

### 2.4. Statistical Analysis

Due to the explorative purpose of this observational study, no sample size calculations were performed; all patients who met entry criteria were included. This study is a retrospective analysis that uses an existing dataset to explore associations and general trends. The focus was on generating insights and hypotheses for future research, rather than on evaluating pre-specified hypotheses with defined error rates.

Descriptive statistics, including mean, standard deviation (SD), and percentages, were used to summarize the demographic characteristics of the study population (age, sex, and BMI) and vitamin D levels. The associations between vitamin D levels, age, BMI, and eGFR were evaluated by Pearson correlation analyses, and the differences in vitamin D levels according to sex were evaluated using the *t*-test for equality of means. The statistical significance of fluctuations in vitamin D levels by month of analysis was evaluated by analysis of variance (ANOVA). The mean vitamin D levels for each month were compared with those for other months with Bonferroni post hoc tests. The normality of vitamin D levels was assessed by the Kolmogorov–Smirnov test. Although this test revealed a statistically significant deviation from normality in vitamin D levels (K-S statistic = 0.096, df = 4979, *p* < 0.001), we proceeded with parametric analyses (such as independent samples *t*-tests and ANOVAs). This decision was justified by the substantial sample size and the interval nature of the data.

To further evaluate variables affecting vitamin D levels, we performed a multiple linear regression analysis with vitamin D levels as the dependent variable. The model was simultaneously adjusted for age (continuous, in years), BMI (continuous, kg/m^2^), eGFR (continuous, mL/min/1.73 m^2^), sex (binary, male/female), and survey month (categorical, January–December).

*p* values < 0.05 were considered statistically significant for all tests. All analyses were two-sided. Statistical analyses were conducted using SPSS for Windows, version 29.0 (IBM Corp., Armonk, NY, USA). GraphPad Prism (Version 10.2.3) and Microsoft Excel (Version 16.8.7) were used to conduct simple analyses.

## 3. Results

### 3.1. Patient Population

A total of 4979 patients met the entry criteria and were included in this observational study. Approximately two-thirds (3230 (64.9%)) were female and the mean (SD) age was 53.6 (15.2) years (Table 2). The mean (SD) BMI was 27.2 (5.6) kg/m^2^, which is generally considered “overweight”. Most patients had acceptable kidney function based on eGFR (normal or mildly decreased; 4634 (93.1%)). However, 345 (6.9%) had eGFR levels < 60 mL/min/1.73 m^2^, indicating moderately to severely decreased kidney function (Table 2). About three-quarters of the cohort (n = 3594 (72.2%)) had a non-inflammatory rheumatology diagnosis and the remainder had various inflammatory rheumatic diseases, most commonly rheumatoid arthritis. Specific diagnoses and their associations with vitamin D levels will be reported separately (manuscript in preparation).

### 3.2. Vitamin D Levels and Status

**Figure 1:**
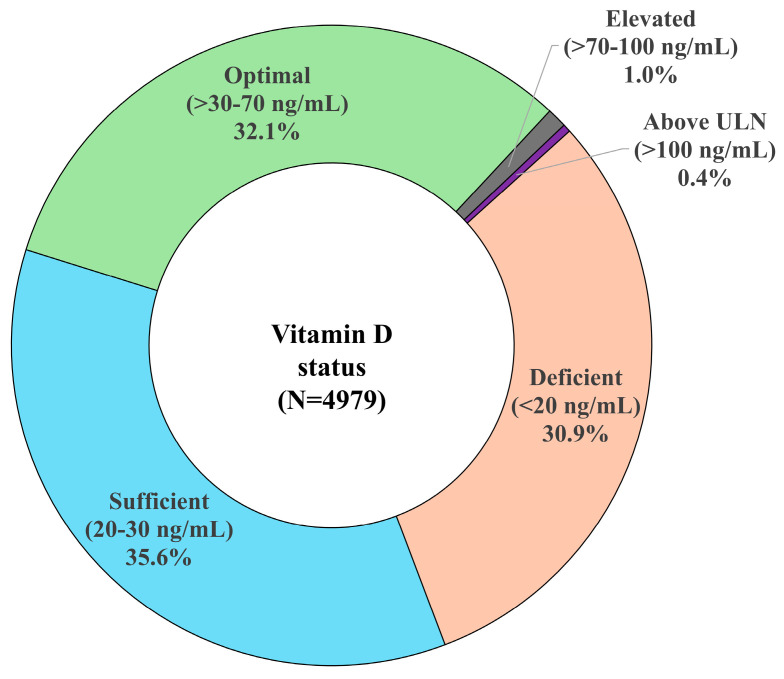
Vitamin D status of clinic patients. ULN, upper limit of normal.

The mean (SD) vitamin D level in this clinic cohort was 27.4 (14.0) ng/mL with a range of 3–240 ng/mL. Both the SD and the range indicate the wide variation in vitamin D levels in this population. With respect to vitamin D status (as defined in Table 1), 30.9% (n = 1540) of the study population had vitamin D levels in the deficient range (<20 ng/mL), 35.6% (n = 1774) had sufficient vitamin D (20 to 30 ng/mL), and 32.1% (n = 1597) had optimal vitamin D levels (>30 to 70 ng/mL) (Figure 1). Only very few patients had vitamin D levels higher than optimal (1.0% [n = 50] with elevated levels >70 to 100 ng/mL and 0.4% [n = 18] with potentially toxic levels above the ULN of >100 ng/mL).

### 3.3. Associations Between Vitamin D Levels and Patient Characteristics

Males had significantly lower mean (SD) vitamin D levels (25.5 (13.6) ng/mL) compared with females (28.4 (14.2)). The mean unadjusted difference between the two populations was −2.9 ng/mL (95% confidence interval (CI) −3.7, −2.1; *p* = 0.001).

Pearson correlation analyses revealed weak, but significant, associations with several patient characteristics. Vitamin D levels were positively correlated with older age (*r* = 0.035; *p* = 0.014), indicating that younger individuals had lower levels of vitamin D, and negatively correlated with BMI (*r* = −0.187; *p* < 0.001), indicating that patients with higher BMI had lower levels of vitamin D. However, age was also positively correlated with BMI (*r* = 0.097, *p* < 0.001), so these analyses may have been influenced by confounding factors. There was also a weak negative correlation between vitamin D levels and higher eGFR levels (*r* = −0.068; *p* < 0.001). Similar results were obtained when the 18 cases with vitamin D levels > 100 ng/mL were excluded from the analyses.

### 3.4. Seasonal Variations in Vitamin D Levels

**Figure 2:**
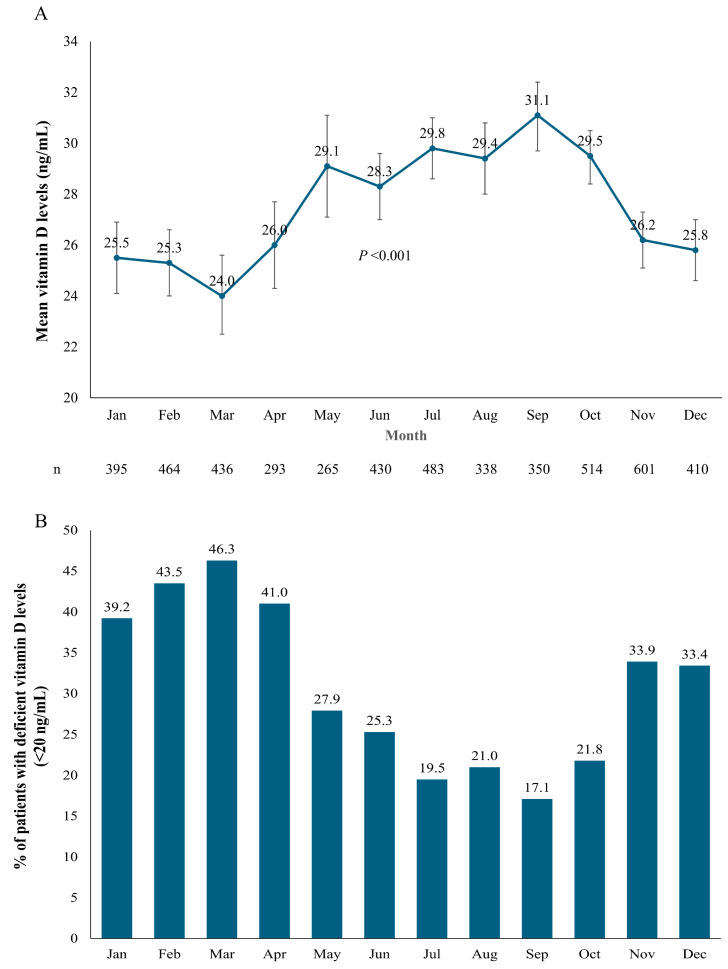
Vitamin D status over the months of the year. (**A**) Mean vitamin D values (error bars indicate the upper and lower 95% confidence interval limits); (**B**) proportion of patients deficient in vitamin D (<20 ng/mL). The numbers of patients tested in each month (n) are shown in the middle of the figure.

The mean vitamin D levels fluctuated over the course of the year, with the lowest levels occurring in the first quarter of the year (January–March) and the highest during the late-spring, summer, and early fall months (May–October) (Figure 2A). This variation was significant according to the ANOVA analyses (*p* < 0.001). The fluctuation in vitamin D levels throughout the year was also reflected in the proportion of patients with deficient vitamin D levels (<20 ng/mL) (Figure 2B). The highest percentages of vitamin D deficiency were observed in January–March, with March having the highest levels of deficiency (46.3%), and the lowest percentages of vitamin D deficiency were observed in July–October, with September having the lowest levels of deficiency (17.1%). There was a 2.7-fold difference between the lowest and highest rates of vitamin D deficiency.

**Figure 3:**
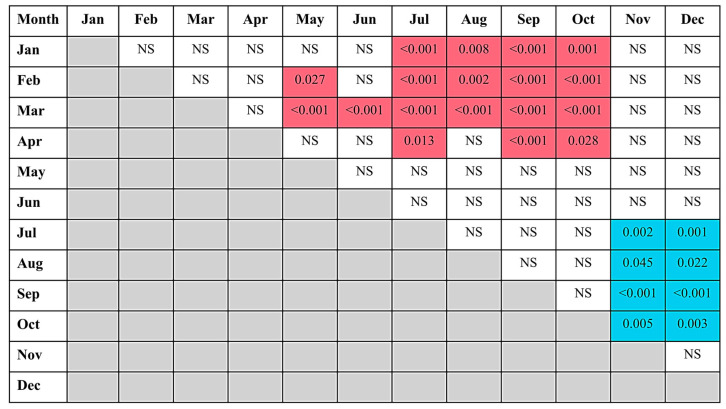
Significant differences between vitamin D levels in different months of the year. The numbers of patients providing data for each month can be found in Figure 2. Mean vitamin D levels for each month were compared with those in the other months with Bonferroni post hoc tests. Red shading indicates that the month shown on the left had significantly lower mean values than the month at the top. Blue shading indicates that the month shown on the left had significantly higher mean values than the month at the top. NS, not significant.

We further evaluated differences between pairs of months (Figure 3). These analyses confirmed the visual observations and indicated that mean vitamin D levels in January through April were, in general, significantly lower than those in July through October, while the mean levels in July through October were significantly higher than those in November and December. Mean vitamin D levels were also significantly lower in February vs. May and in March vs. May and June.

### 3.5. Regression Analysis of Variables Influencing Vitamin D Levels

To comprehensively assess the relationships between vitamin D levels and key variables, we conducted a multiple linear regression analysis with serum vitamin D levels as the dependent variable and the independent variables of age, BMI, eGFR, sex, and survey month. The regression analysis confirmed that BMI (*p* < 0.001) and eGFR (*p* < 0.001) were negatively associated with vitamin D levels and that male sex (*p* < 0.001) also predicted lower values. Several summer and autumn months were associated with significantly higher vitamin D levels compared with January (May–October; all *p* ≤ 0.002 with most *p* < 0.001). Overall, the model explained a small, but significant, portion (7.5%; R^2^ = 0.075) of the variance in vitamin D levels (*F* = 26.64; df = 15; *p* < 0.001).

## 4. Discussion

In this retrospective observational study of EHR data from patients who were referred for the evaluation of rheumatologic complaints, approximately 30% of patients were deficient in vitamin D (<20 ng/mL) and only about one-third (32.1%) had vitamin D levels considered optimal (>30 to 70 ng/mL) by the majority of guidelines [21] (see Table 1 for definitions used in this study). The percentage of patients with levels indicating vitamin D deficiency increased to over 40% during winter/early spring months, with a high of 46.3% in March. Vitamin D levels were significantly lower in males, younger individuals, individuals with higher BMI or higher eGFR, and during the winter months. These findings confirm the relevance of testing vitamin D levels and suggest opportunities for intervention with counseling and vitamin D supplementation in clinical cohorts of rheumatology patients. The cohort in this study was highly heterogeneous with respect to diagnosis and a minority had an inflammatory rheumatic condition. It is thus likely that the observations here are applicable to other clinical populations as well.

Although the level of vitamin D deficiency in this population was high enough to raise clinical concern, it was only approximately half that reported for the German adult population as a whole (56%) [9,10]. There are several possible explanations for this discrepancy, including different assay systems, the higher proportion of males in the population study (48.0% vs. 35.1% in the study reported here), and the fact that the majority of individuals in the population study (70.6%) resided in latitudes north of the location in our study center (48° N), thereby reducing their sun exposure and increasing the risk of vitamin D deficiency. In addition, individuals in the population study reported a very low use of vitamin D supplementation (3.8%). Our database did not contain information on vitamin D supplementation, which is an important limitation of this study. However, it is possible that a clinical population would have higher levels of vitamin D usage than the overall general population. The prevalence of vitamin D deficiency in our study is similar to the 33.9% reported in a German study of patients preparing to undergo arthroplasty [22], but lower than observed in other clinical populations in Germany [23,24]. It should be noted, however, that comparisons between community samples and other clinical populations may be influenced by selection bias and differences in vitamin D assays.

In our study, the correlations between vitamin D levels and patient characteristics were weak (*r* < 0.2) and the applied multiple regression model only explained 7.5% of the variation in vitamin D levels, likely reflecting the diversity in the clinical population and the impact of additional factors affecting vitamin D levels, such as sun exposure, disease activity, medications, skin tone, and others of the many factors that affect vitamin D levels. The statistical significance observed for some results may be due to sample size, rather than to a clinically relevant association. However, the consistent direction of findings across both correlation and multiple linear regression analyses suggests the presence of clinically meaningful associations, even though the effect sizes, as indicated by the magnitudes of the correlation coefficients and standardized beta weights, were numerically modest. As has been observed in other studies [11,22,24], a higher BMI was associated with lower vitamin D levels in our analyses. The reduced vitamin D levels in patients with higher BMIs seems somewhat counterintuitive, but may be due to the sequestration of vitamin D into adipose tissue [14]. We also found that younger individuals had lower levels of vitamin D, which is consistent with recent studies in the US [11,25]. One of these studies, which was based on data from the National Health and Nutrition Examination Survey (NHANES) and included 31,628 participants from 2011 to 2018, found that vitamin D levels were distributed in a U-shaped pattern, with individuals <4 years of age and ≥60 years of age having the highest values [11]. Because our cohort did not include patients under 11 years of age, the influence of higher vitamin D levels at very young ages was not a factor in our analysis, making it more likely that we would observe an association between younger adult patients and lower levels of vitamin D. An association of male gender with reduced vitamin D levels has also been observed [11,25], but this observation is somewhat inconsistent across studies [26]. Vitamin D levels are known to be influenced by sex hormones, including testosterone, which inhibits vitamin D metabolism [26].

Lower vitamin D levels were weakly associated with higher eGFR levels (improved kidney function). It is likely that this finding is not clinically meaningful and solely due to the large sample size. However, a similar association was observed in a large study of individuals in the US National Health and Nutrition Examination Survey database [27]. These results can potentially be explained by the role of the kidney in converting 25(OH)D to the active form of vitamin D, 1,25(OH)_2_D, which may be a more accurate marker of vitamin D deficiency in patients with renal dysfunction [28].

As in our study, seasonal variations in vitamin D levels have been consistently observed; a recent meta-analysis found that the risk of vitamin D deficiency was 1.7-fold higher in winter/spring than in summer/autumn [13]. These figures are in line with the 2.7-fold difference between the highest (March) and lowest (July) vitamin D deficiency rates observed in our study. The lower levels of vitamin D during winter months are probably mostly driven by differences in sun exposure during the course of the year, both due to changes in the solar zenith angle and to alterations in individual sun exposure during periods of inclement weather [14]. In our study, the lowest mean vitamin D levels were observed in November through April; these months also had the highest percentages of patients with vitamin D deficiency. Educating patients on seasonal changes in vitamin D levels may help to alter this pattern.

Given the high levels of vitamin D deficiency observed in our cohort and throughout Germany, supplementation with vitamin D may be beneficial for some patients. Guidelines concerning vitamin D supplementation in adults vary widely in their recommendations [21]. The German Osteology Association guidelines do not state a target vitamin D level, but recommend that postmenopausal women and men 50 years of age or older be supplied with vitamin D at a minimum amount of 800 International Units (20 µg)/day through diet or sunlight exposure, unless medically contraindicated [29]. The German Nutrition Society considers serum vitamin D concentrations of ≥20 ng/mL (50 nmol/L) to be the target level and recommends that a vitamin D intake of 20 µg/day be ensured in individuals in whom endogenous vitamin D production is lacking, such as in older individuals or those who are seldom exposed to the sun [30]. The Endocrine Society, which published updated guidelines in 2024, did not find evidence to support a target serum level for vitamin D, but recommends empiric vitamin D supplementation for those aged 1 to 18 years and over 75 years of age and those who are pregnant or have a high risk of diabetes [31]. In patients with renal dysfunction, observational studies suggest that vitamin D supplementation may help to improve a wide range of outcomes [32,33,34]. However, clinical trials of vitamin D supplementation in this population have not shown a clear benefit, and recommendations on the use of vitamin D in the management of patients with renal impairment are currently mixed [32,33,34].

With respect to rheumatology patients, the American College of Rheumatology [35] and the European League Against Rheumatism [36] recommend vitamin D supplementation in patients receiving glucocorticoid therapy, but do not provide guidance for the use of vitamin D in other rheumatology patient populations. In our practice, we try to ensure that patient vitamin D levels are at least within the sufficient range. If the patient is receiving concomitant glucocorticoids, supplementation is then recommended with the goal of achieving vitamin D levels within the optimal range (see Table 1).

As mentioned previously, there is mixed evidence concerning the benefits of vitamin D supplementation for non-skeletal diseases [1,37]. Nevertheless, the current suggestive body of evidence, combined with the observed reduction of mortality in elderly individuals who take vitamin D supplements [8] and the overall safety of vitamin D supplements [5], suggest that augmentation of vitamin D levels should be considered for some patients.

The limitations of this study include its retrospective, observational nature and the use of cohort time points, rather than longitudinal analysis, to evaluate seasonal variations in vitamin D levels. Our database did not contain information on vitamin D supplementation or nutritional variables, such as milk intake; these factors are known to affect vitamin D levels [7,11,25]. We acknowledge that different experts and guidelines support varying levels for vitamin D deficiency and optimal levels [21]. Alterations in the cut-offs used here would impact our assessments of the proportions of patients for different categories of vitamin D status. Our study was based on measurements of 25(OH)D. Analyses of the active form of vitamin D, 1,25(OH)_2_D, may have provided additional information on vitamin D status, particularly in patients with renal impairment, granulomatous disorders, or rare genetic conditions [4,5].

## 5. Conclusions

In conclusion, the high proportion of patients with vitamin D levels indicating a vitamin D deficiency suggests opportunities for patient education to improve vitamin D status, particularly in patients who are male, younger, or have an elevated BMI. All patients should also be made aware of the strong seasonal effects on vitamin D levels associated with reduced sun exposure.

## Figures and Tables

**Table 1:** Cut-offs used for vitamin D status categories.

Status Category	Vitamin D Level	
	ng/mL	nmol/L
Deficient	<20	<50
Sufficient	20–30	50–75
Optimal	>30–70	>75–175
Elevated	>70–100	>175–250
Above ULN	>100	>250

ULN, upper limit of normal.

**Table 2:** Demographic and clinical characteristics of the clinic cohort.

Variable	Clinic Cohort (N = 4979)
Sex, n (%)	
Females	3230 (64.9%)
Males	1749 (35.1%)
Age, mean (SD) [range] years	53.6 (15.2) [11–94]
Body mass index, mean (SD) [range] kg/m^2^	27.2 (5.6) [15.4–67.1]
Renal status as assessed by eGFR ^a^, n (%)	
≥90 (normal or high kidney function)	2559 (51.4%)
60–89 (mildly decreased kidney function)	2075 (41.7%)
30–59 (moderately decreased kidney function)	321 (6.4%)
15–29 (severely decreased kidney function)	20 (0.4%)
<15 (kidney failure)	4 (0.1%)
Vitamin D, mean (SD) [range] ng/mL	27.4 (14.0) [3–240]

^a^ In mL/min/1.73 m^2^ adjusted for age and sex. eGFR, estimated glomerular filtration rate; SD, standard deviation.

## Data Availability

The original contributions presented in this study are included in the article. Further inquiries can be directed to the corresponding author.

## Abbreviations

The following abbreviations are used in this manuscript:

ANOVA	Analysis of variance
BMI	Body mass index
CKD	Chronic kidney disease
eGFR	Estimated glomerular filtration rate
EHR	Electronic health record
NS	Not significant
SD	Standard deviation

## References

[1] Rebelos E., Tentolouris N., Jude E. (2023). The role of vitamin D in health and disease: A narrative review on the mechanisms linking vitamin D with disease and the effects of supplementation. Drugs.

[2] Charoenngam N., Holick M.F. (2020). Immunologic effects of vitamin D on human health and disease. Nutrients.

[3] Garnett E., Li J., Rajapakshe D., Tam E., Meng Q.H., Devaraj S. (2019). Efficacy of two vitamin D immunoassays to detect 25-OH Vitamin D2 and D3. Pract. Lab. Med..

[4] Alonso N., Zelzer S., Eibinger G., Herrmann M. (2022). Vitamin D metabolites: Analytical challenges and clinical relevance. Calcif. Tissue Int..

[5] Giustina A., Bilezikian J.P., Adler R.A., Banfi G., Bikle D.D., Binkley N.C., Bollerslev J., Bouillon R., Brandi M.L., Casanueva F.F. (2024). Consensus statement on vitamin D status assessment and supplementation: Whys, whens, and hows. Endocr. Rev..

[6] National Institutes of Health (2024). Vitamin D Fact Sheet for Health Professionals. https://ods.od.nih.gov/factsheets/VitaminD-HealthProfessional/.

[7] Rupprecht M., Wagenpfeil S., Schöpe J., Vieth R., Vogt T., Reichrath J. (2023). Meta-analysis of European clinical trials characterizing the healthy-adult serum 25-hydroxyvitamin D response to vitamin D supplementation. Nutrients.

[8] Bjelakovic G., Gluud L.L., Nikolova D., Whitfield K., Wetterslev J., Simonetti R.G., Bjelakovic M., Gluud C. (2014). Vitamin D supplementation for prevention of mortality in adults. Cochrane Database Syst. Rev..

[9] Rabenberg M., Scheidt-Nave C., Busch M.A., Rieckmann N., Hintzpeter B., Mensink G.B.M. (2015). Vitamin D status among adults in Germany—Results from the German Health Interview and Examination Survey for Adults (DEGS1). BMC Public Health.

[10] Rabenberg M., Scheidt-Nave C., Busch M.A., Thamm M., Rieckmann N., Durazo-Arvizu R.A., Dowling K.G., Škrabáková Z., Cashman K.D., Sempos C.T. (2018). Implications of standardization of serum 25-hydroxyvitamin D data for the evaluation of vitamin D status in Germany, including a temporal analysis. BMC Public Health.

[11] Subramanian A., Burrowes H.B., Rumph J.T., Wilkerson J., Jackson C.L., Jukic A.M.Z. (2024). Vitamin D levels in the United States: Temporal trends (2011–2018) and contemporary associations with sociodemographic characteristics (2017–2018). Nutrients.

[12] (2011). CaMos Research Group; Greene-Finestone, L.S.; Berger, C.; De Groh, M.; Hanley, D.A.; Hidiroglou, N.; Sarafin, K.; Poliquin, S.; Krieger, J.; Richards, J.B.; et al. 25-hydroxyvitamin D in Canadian adults: Biological, environmental, and behavioral correlates. Osteoporos. Int..

[13] Cui A., Zhang T., Xiao P., Fan Z., Wang H., Zhuang Y. (2023). Global and regional prevalence of vitamin D deficiency in population-based studies from 2000 to 2022: A pooled analysis of 7.9 million participants. Front. Nutr..

[14] Tsiaras W., Weinstock M. (2011). Factors influencing vitamin D status. Acta Derm. Venereol..

[15] El-Gabalawy H., Guenther L.C., Bernstein C.N. (2010). Epidemiology of immune-mediated inflammatory diseases: Incidence, prevalence, natural history, and comorbidities. J. Rheumatol. Suppl..

[16] GBD 2021 Osteoarthritis Collaborators (2023). Global, regional, and national burden of osteoarthritis, 1990-2020 and projections to 2050: A systematic analysis for the Global Burden of Disease Study 2021. Lancet Rheumatol..

[17] Charoenngam N. (2021). Vitamin D and rheumatic diseases: A review of clinical evidence. Int. J. Mol. Sci..

[18] Inker L.A., Eneanya N.D., Coresh J., Tighiouart H., Wang D., Sang Y., Crews D.C., Doria A., Estrella M.M., Froissart M. (2021). New creatinine- and cystatin C–based equations to estimate GFR without race. N. Engl. J. Med..

[19] Stevens P.E., Ahmed S.B., Carrero J.J., Foster B., Francis A., Hall R.K., Herrington W.G., Hill G., Inker L.A., Kazancıoğlu R. (2024). KDIGO 2024 clinical practice guideline for the evaluation and management of chronic kidney disease. Kidney Int..

[20] Roche Diagnostics (2025). Elecsys Vitamin D Total III. https://elabdoc-prod.roche.com/eLD/api/downloads/8e91504f-e2dc-ef11-2891-005056a772fd?countryIsoCode=XG.

[21] Dai Z., McKenzie J.E., McDonald S., Baram L., Page M.J., Allman-Farinelli M., Raubenheimer D., Bero L.A. (2021). Assessment of the methods used to develop vitamin D and calcium recommendations—A systematic review of bone health guidelines. Nutrients.

[22] Heinz T., Hoxha M., Anderson P.M., Jakuscheit A., Weißenberger M., Lüdemann M., Rak D., Rudert M., Horas K. (2024). Prevalence and associated risk factors for hypovitaminosis D in patients scheduled for primary total knee arthroplasty in Germany. Nutrients.

[23] Zittermann A., Zelzer S., Herrmann M., Kleber M., Maerz W., Pilz S. (2025). Association between magnesium and vitamin D status in adults with high prevalence of vitamin D deficiency and insufficiency. Eur. J. Nutr..

[24] Mamilos A., Matzaroglou C., Maier G.S., Zawy Alsofy S., Drees P., Kafchitsas K. (2023). Vitamin D deficiency in orthopedic patients in different latitudes—First study comparing German and Greek populations. Osteology.

[25] Liu X., Baylin A., Levy P.D. (2018). Vitamin D deficiency and insufficiency among US adults: Prevalence, predictors and clinical implications. Br. J. Nutr..

[26] Wierzbicka A., Oczkowicz M. (2022). Sex differences in vitamin D metabolism, serum levels and action. Br. J. Nutr..

[27] Geng J., Qiu Y., Li Y., Li J., Liao R., Du H., Jiang L., Wang L., Qin Z., Yang Q. (2022). Associations between 25-hydroxyvitamin D, kidney function, and insulin resistance among adults in the United States of America. Front. Nutr..

[28] De Boer I.H., Sachs M.C., Chonchol M., Himmelfarb J., Hoofnagle A.N., Ix J.H., Kremsdorf R.A., Lin Y.S., Mehrotra R., Robinson-Cohen C. (2014). Estimated GFR and circulating 24,25-dihydroxyvitamin D3 concentration: A participant-level analysis of 5 cohort studies and clinical trials. Am. J. Kidney Dis..

[29] Wissenschaftlicher Dachverband Osteologie—DVO (Scientific Umbrella Association for Osteology) (2023). Prophylaxe, Diagnostik und Therapie der Osteoporose Bein Postmenopausalen Frauen und bei Männern ab Dem 50.Lebensjahr. https://leitlinien.dv-osteologie.org/wp-content/uploads/2024/02/DVO-Leitlinie-zur-Diagnostik-und-Therapie-der-Osteoporose-Version-2.1.-2023-002.pdf.

[30] German Nutrition Society (2012). New reference values for vitamin D. Ann. Nutr. Metab..

[31] Demay M.B., Pittas A.G., Bikle D.D., Diab D.L., Kiely M.E., Lazaretti-Castro M., Lips P., Mitchell D.M., Murad M.H., Powers S. (2024). Vitamin D for the prevention of disease: An Endocrine Society clinical practice guideline. J. Clin. Endocrinol. Metab..

[32] Yeung W.-C.G., Toussaint N.D., Badve S.V. (2024). Vitamin D rherapy in chronic kidney disease: A critical appraisal of clinical trial evidence. Clin. Kidney J..

[33] Jørgensen H.S., Vervloet M., Cavalier E., Bacchetta J., De Borst M.H., Bover J., Cozzolino M., Ferreira A.C., Hansen D., Herrmann M. (2025). The role of nutritional vitamin D in chronic kidney disease–mineral and bone disorder in children and adults with chronic kidney disease, on dialysis, and after kidney transplantation—A European consensus statement. Nephrol. Dial. Transplant..

[34] Vervloet M.G., Hsu S., De Boer I.H. (2023). Vitamin D supplementation in people with chronic kidney disease. Kidney Int..

[35] Humphrey M.B., Russell L., Danila M.I., Fink H.A., Guyatt G., Cannon M., Caplan L., Gore S., Grossman J., Hansen K.E. (2023). 2022 American College of Rheumatology guideline for the prevention and treatment of glucocorticoid-induced osteoporosis. Arthritis Rheumatol..

[36] Duru N., Van Der Goes M.C., Jacobs J.W.G., Andrews T., Boers M., Buttgereit F., Caeyers N., Cutolo M., Halliday S., Da Silva J.A.P. (2013). EULAR evidence-based and consensus-based recommendations on the management of medium to high-dose glucocorticoid therapy in rheumatic diseases. Ann. Rheum. Dis..

[37] Liu D., Meng X., Tian Q., Cao W., Fan X., Wu L., Song M., Meng Q., Wang W., Wang Y. (2022). Vitamin D and multiple health outcomes: An umbrella review of observational studies, randomized controlled trials, and Mendelian randomization studies. Adv. Nutr..

